# Quantitative Proteomic Analysis of Gene Regulation by miR-34a and miR-34c

**DOI:** 10.1371/journal.pone.0092166

**Published:** 2014-03-17

**Authors:** Olivia A. Ebner, Matthias Selbach

**Affiliations:** Max Delbrueck Center for Molecular Medicine, Berlin, Germany; University of Nebraska Medical Center, United States of America

## Abstract

microRNAs (miRNAs) repress target genes by destabilizing mRNAs and/or by inhibiting translation. The best known factor for target recognition is the so called seed – a short continuous region of Watson-Crick base pairing between nucleotides 2–7 of the miRNA and complementary sequences in 3′ untranslated regions of target mRNAs. The miR-34 family consists of three conserved members with important tumor suppressor functions linked to the p53 pathway. The family members share the same seed, raising the question if they also have the same targets. Here, we analyse the effect of miR-34a and miR-34c on protein synthesis by pSILAC. Despite significant overlap, we observe that the impact of both family members on protein synthesis differs. The ability to identify specific targets of a family member is complicated by the occurrence of * strand mediated repression. Transfection of miR-34 chimeras indicates that the 3′end of the miRNA might be responsible for differential regulation in case of targets without a perfect seed site. Pathway analysis of regulated proteins indicates overlapping functions related to cell cycle and the p53 pathway and preferential targeting of several anti-apoptotic proteins by miR-34a. We used luciferase assays to confirm that Vcl and Fkbp8, an important anti-apoptotic protein, are specifically repressed by miR-34a. In summary, we find that miR-34a and miR-34c down-regulate distinct subsets of targets which might mediate different cellular outcomes. Our data provides a rich resource of miR-34 targets that might be relevant for clinical trials that want to implement the miR-34 family in cancer therapy.

## Introduction

Animal microRNAs (miRNAs) are a class of small endogenous, non-coding RNAs mediating posttranscriptional gene silencing [1], [2]. miRNAs have a widespread impact on regulation of gene expression and evolution and are thought to affect over 50% of all human genes [2], [3], [4], [5]. Their function is not restricted to normal organism development: miRNAs also play a vital role in diseases such as cancer, where they can act as oncogenes or tumor suppressors [6], [7].

miRNAs are transcribed as longer hairpin molecules that are processed over several steps until they are cut by DICER into duplexes of their final 22–23nt length [8]. As a last step, one strand of the miRNA duplex (“mature strand”) is incorporated into the RNA-induced silencing complex (RISC) while the other, so called star (*) strand is supposedly degraded [9]. Once integrated into the RISC miRNAs repress target mRNAs via either direct mRNA cleavage or translational regulation associated with mRNA degradation [2], [10], [11]. The overall role of mRNA degradation and translational repression for miRNA-mediated regulation is not entirely clear.

One of the most important questions is how miRNAs recognize their target mRNAs. The best understood factor for target recognition are so-called “seed” sites: stretches of perfect Watson-Crick base pairing between nucleotide 2–7 of the miRNA and complementary sequences in the 3′ untranslated region (3′UTR) of target mRNAs. The correlation between target repression and 3′UTR seeds had been found early in the exploration of miRNAs [12], [13]. While the seed is generally considered to be the most important sequence feature for target recognition, it is important to note that it is neither necessary nor sufficient. For example, some miRNA targets are down-regulated despite missing a complete seed match [14]. Others are dependent on so called “centered” seeds spanning miRNA nucleotides 4 to 15 [15]. Finally, many mRNAs which contain a 3′UTR seed match are not repressed by over-expression of the corresponding miRNA. Collectively, these observations indicate that the seed is not the only factor involved in target recognition.

Since members of miRNAs families usually share the same seed site but differ in their remaining sequence they present a natural setup to study target selection independent of seed differences [16], [17]. Differential targeting of family members should be mediated by variations aside the seed site and be more physiological than artificial mutations of miRNAs. In fact, it has been proposed that miRNA families do have different targets depending on their 3′end sequence [14]. However, only few studies investigated target selection of miRNA families by over-expression of individual family members so far. Two microarray studies on the miR-16 and miR-34 families came to the conclusion that members of both families show functional redundancy [18], [19]. The miR-34 family is a particularly interesting example as one of the few families that are also conserved in *Drosophila* and *C. elegans* [20]. While invertebrates only possess one miR-34 gene, the miR-34 family consists of three members in vertebrates encoded at two different gene loci [21], [22]. While miR-34a and miR-34c are perfectly conserved in sequence between human, mouse and chicken, miR-34b shows slight nucleotide alterations between the three species [23].

The miR-34 family is part of the p53 stress and DNA damage response pathway and has widespread regulatory effects on the cell [24]. Activation of p53 by genotoxic stress activates expression of miR-34 family members [19], [23]. In turn, miR-34a has been shown to up-regulate p53 activity via a positive feedback loop involving Sirtuin 1 leading to apoptosis [25]. Several targets of the miR-34 family mediate cell progression and block apoptosis, suggesting that by repressing these targets miR-34 acts as a tumor suppressor [22], [24], [26], [27], [28], [29]. Validated targets include Cdk4, Cdk6, Hmga2, c-Met and Akt. Most of these targets have been validated for miR-34a while the two other family members are less well studied. Interestingly, despite the obvious links between the miR34 family and p53, recent *in vivo* studies showed that mice lacking all family members have normal p53-dependent responses [30], [31]. Ectopic expression of miR-34 within mouse tumor models, however, can significantly reduce tumor growth in mice and treatment with miR-34 is currently even considered for clinical trials [32].

Whether different members of the miR-34 family have different targets is still an open question. Despite the fact that differential targeting between the miR-34 members has been reported, recent reviews of the miR-34 family come to the conclusion that they are redundant in function [27], [28]. However, so far studies mainly focused only on mRNA levels or individual selected targets. These approaches cannot cover the effect of differential targeting miRNA family members at the protein level on a global scale. Studies have shown that the degree of translational repression by miRNAs can amount to a large part of regulation [3], [11]. In fact, some specific targets of the miR-34 family such as c-Myc have been shown to be only translationally repressed [33]. Therefore, differences between family members may only become apparent at the protein level.

We developed pulsed stable isotope labeling by amino acids in cell culture (pSILAC) to quantify relative changes in protein synthesis on a global scale [3], [34]. pSILAC has since been applied to assess translational regulation in several examples, including regulation by miR-34a [29], [35]. Here, we combine pSILAC and mRNA quantification by microarray to assess the effect of miR-34a and miR-34c on gene expression in HeLa cells. We focused on these two members since they show the biggest differences in sequence and conserved from chicken to human while miR-34b shows some sequence divergence between these species [23]. In addition, we also generated artificial chimeras between miR-34a and miR-34c to assess if target specificity depends on the 5′ or 3′ end. While we found considerable overlap, our results also indicate that both family members target distinct subsets of genes, suggesting non-redundant cellular functions.

## Materials and Methods

### miRNA design

Fully complement siRNA duplexes for miR-34 members and chimeras were purchased from Dharmacon in annealed, desalted and 2′-deprotected form for direct use. Full complement duplexes were designed as follows (sense and antisense 5′-3′):

miR-34a: UGGCAGUGUCUUAGCUGGUUGU/ AACCAGCUAAGACACUGCCAUA

miR-34c: AGGCAGUGUAGUUAGCUGAUUGC/ AAUCAGCUAACUACACUGCCUGG

miR-34ac: UGGCAGUGUAGUUAGCUGAUUGC/ AAUCAGCUAACUACACUGCCAUA

miR-34ca: AGGCAGUGUCUUAGCUGGUUGU/ AACCAGCUAAGACACUGCCUGG

### Cell culture and Transfection of HeLa cells with double-stranded RNAs

HeLa (LGC Promochem) cells for mass spectrometry experiments were grown at 37°C with 5% CO2 in Dulbecco’s Modified Eagle’s Medium (DMEM) High Glucose (4.5 g/l) (PAA, custom preparation) supplemented with 10% sterile-filtered dialyzed fetal bovine serum (dFBS, Sigma-Aldrich), 4 mM stable Glutamine (l-alanyl-l-glutamine, PAA), light L-arginine (84 mg/l) and L-lysine (40 mg/l) [36]. The cells were transfected and processed as described before [3]. In short, transfection of synthetic RNAs (Dharmacon) of a final concentration of 100 nM was done according to the manufacturers protocol using DharmaFECT1 (Dharmacon). For transfection HeLa cells were plated on 10 cm dishes in antibiotic-free light (L) SILAC medium at a confluence of ∼70–80%. A mock control transfected with ddH_2_O instead of RNA was prepared for each RNA transfected sample. 8h after transfection, cells were washed twice and the medium for RNA transfected samples was changed to medium-heavy (M) SILAC medium (84 mg/l ^13^C_6_-L-arginine and 40 mg/L ^2^H_4_-L-lysine), while mock transfections were transferred to heavy (H) SILAC medium (84 mg/l ^13^C_6_^15^N_4_-L-arginine and 40 mg/l ^13^C_6_^15^N_2_-L-lysine). 24h after pulse labeling cells were scraped off the plates, combined with the matching control, lysed using RIPA buffer and subjected to one-dimensional SDS-PAGE as described below. In addition to the original miR-34a and miR-34c transfection experiment, two replicates of miR-34a and one replicate of miR-34c were done in an independent transfection as were the miR-34 chimera RNA transfections.

### Determination of transfection efficiency

To ensure delivery of our synthetic siRNA duplexes we did transfections of double stranded, fluorescently labeled RNA oligomers (“BLOCK-IT”, Invitrogen) prior to further transfection experiments. The oligomers were transfected as described above. 8h after transfection cells were washed with 1x PBS (Gibco) and fixated in 4% paraformaldehyde (PFA) in D-PBS. Transfection efficiency was compared via the fluorescence of transfected versus non-transfected cells on a fluorescence microscopy (Keyence Biozero).

### SDS-PAGE and tryptic digestion of samples

About 100 μg of mixed protein samples were loaded on a 4–12% NuPage™ Bis-Tris gradient gels (Invitrogen) and separated according to the manufacturer’s instructions. Gels were subjected to fixative solutions and colloidal Coomassie Brilliant Blue G-250 (Invitrogen) and single protein lanes were subsequently cut into 12-15 slices. Destaining, washing and tryptic digestion was done as described before [37]. Before mass spectrometry samples were extracted and desalted using StageTips [38].

### LC-MS/MS measurement

LC – MS/MS analysis was performed as described before [3]. Peptides were analyzed using online reversed-phase liquid chromatography (rpHPLC) connected to an electrospray ion source (Proxeon) of a LTQ-Orbitrap mass spectrometer. rpHPLC was done using either the Agilent HPLC 1200 or Eksigent NanoLC – 1D Plus system. miR-34a and miR-34c samples were measured on Orbitrap classic and XL instruments while the chimera samples (miR-34ac and miR-34ca) were analysed on an Orbitrap Velos (Thermo Fisher). For HPLC separation we used fritless C18 microcolumns (75 m ID packed with ReproSil-Pur C18-AQ 3-µm resin, Dr. Maisch GmbH), manually produced as describe before [39]. Peptide were loaded onto the column using a flow rate of 500 nl/min (Agilent HPLC 1200) or 250 nl/min (Eksigent/Proxeon HPLC). Gradients were run and subsequently eluted with a flow rate of 200 nl/min with a 10 to 60 % acetonitrile gradient of 155min or 240min in 0.5% acetic acid. The Orbitraps were operated in a top5 or 10 mode using data dependent acquisition of MS/MS scans as essentially described before [40]. In this mode, every full MS scan in the Orbitrap (*m*/*z* 300–1700; resolution 60,000; target value 1×10^6^) is followed up by 5 or 10 consecutive MS/MS scans in the LTQ isolating and fragmenting the 5 or 10 top most intense ions (charge > 1; target value 5000; monoisotopic precursor selection enabled) by collision induced dissociation (CID; 35% normalized collision energy and wideband activation enabled). Dynamic exclusion of 60sec was used to minimize repeated fragmentation of the same ions.

### Processing of MS data

Mass spectrometry data were processed using the MaxQuant software version 1.0.13.13 [41] using the MASCOT search engine (version 2.2, MatrixScience). To facilitate data integration all raw files were processed together. Labels were set to medium-heavy (Arg6 and Lys4) and heavy (Arg10 and Lys8) with a maximum of three labeled amino acids per peptide (top 6 MS/MS peaks per 100 Da; Quant.exe). The resulting peak lists were submitted to the MASCOT engine and searched for matches with an in-house curated concatenated target-decoy database consisting forward and reversed proteins (supplemented with a fasta file for identification of common contaminants). Version 3.64 of the human IPI database (84,054 entries) was used for our analysis. Tryptic specificity with a maximum of two missed cleavages was required. The mass tolerance was set to 0.5 Da for fragment ions. For precursor ions, individual mass tolerances were assigned by MaxQuant as described [41]. Accepting thresholds for individual spectra were defined based on the target decoy database search strategy implemented in the MaxQuant software. Variable modifications were set to oxidation of methionine and acetylation of the protein N-terminus, while carbamidomethylation of cysteine was selected as fixed modification. For protein assembly only peptides with a minimum length of 6 amino acids were considered and per protein group at least one peptide was required. A maximum false discovery rate (FDR) of 1% (peptide and protein level) was allowed which was calculated by matches to reversed sequences in the concatenated target-decoy database. Only unique and “razor” peptides (non-unique peptides of to the protein group with the highest number of peptides) with a minimum ratio count of two were used for protein quantification. Normalization of data was done by MaxQuant under the assumption that most protein ratios do not change upon miRNA transfection. After removal of reverse hit and contaminants, we matched Reseq NP identifier of the MaxQuant output table with a list of Refseq NM IDs containing the number of mature or *seed sites in the 3′UTR of the respective gene. This list was curated using a list of human gene 3′UTR sequences downloaded from the UCSC Genome Browser (http://genome.ucsc.edu, gene list update from February 2009). This list of 3′UTRs was also the basis for all further studies (Sylarray, Sequence motifs analyses). The script also mapped PicTar (http://pictar.bio.nyu.edu/cgibin/new_PicTar_mouse.cgi) predictions for all miR-34 members to our protein data. As a last step, log2 fold changes were calculated from the normalized H/M ratios of each sample. The resulting table was merged with the microarray data.

### Luciferase cloning and assays

The 3′UTRs of Fkbp8 (NM_012181) and Prkar2a (NM_004157) were synthesized and cloned into the pRL-TK Cxcr4 vector with prior removal of the Cxcr4 4x target site by Not1/Xho1 digestion and verified by sequencing (SINA Science Services GmbH). The Vcl (NM_014000) vector was a kind gift of Dr. Markus Kaller cloned into the pGL3-control-MCS vector [29]. As positive control we used the known target of miR-34a c-Met (NM_000245) from previous studies of our laboratory [3].

The sequence of the Fkbp8 3′UTR without poly-A signal used for cloning is:

5′CCACCTAGGTGGCTGCCACCCCCTCTGCACACCATGGACCCTGCCCTGCGCTCCCCAACTCCCCCAGGCTCCCTGTCCACTGCCCTCCCTGGTCTGGCCCCCTCCTCCGGGTTAGGGGAGCAAGGATTGGGGGTCGTGCAGCCCAGCCAGCAGGAGGGACTGAGGCCCTCTAGGAGGAAAGCCCAGAGGGAGGGGGCCCTCATTCCTTCAGACCCAGTTTTCCCCCACCCTCCTTACCCCGCTGGGCTAGGTCTCCGCCAGGGCTGGCCTCAGTTTCTCCTCAACAGGCCTGGGGGCAGCCCTTCCCCTGCCTAGTCCCCGCCTGAGTGCCAGCCCCCCACCCCGCCTGCCGCCCCCTGTCCAGGTTCCCTCCCCGCCACAGTGAAATAAAGCATCCCACCCTGCAGTTTĆ3

The sequence of the Prkar2a 3′UTR without poly-A signal used for cloning is:

5′GTGTGCCACACCCCAGAGCCTTCTTAGTGTGACACCAAAACCTTCTGGTCAGCCACAGAACACATACAGAAAACAGACATGACAGAACTGTTCCTGCCGTTGCCGCCACTGCTGCCATTGCTGTGGTTATGGGCATTTAGAAAACTTGAAAGTCAGCACTAAAGGATGGGCAGAGGTTCAACCCACACCTCCACTTTGCTTCTGAAGGCCCATTCATTAGACCACTTGTAAAGATTACTCCAACCCAGTTTTTATATCTTTGGTTCAAAACGGCATGTCTCTCCAACAATTTAAGTGCCTGATACAAAGTCCAAAGTATAAACATGCTCCTTTCCTCTCTTGCTGCTACTCTTGCTTTTGGAAGTTACCACAGGGTCTGCAGAAACCTGTTGTATAACTGTAGACACTCTCTAATGGTTCTCAAAGGAGGAAATGTAGCCTTCAGTCTCCTCATTTGTCCTTTGAGGAAGTCCACATTTGTTCACAGTTGCAGCCTTTGGTTTTACAGTGGGAAATGGTGGTGGATGATATGGACATATGTAGCCCAGTGGCATTGTACTTTCTGCTGACAGCTGCACACATTACAGCTGTCTCCAAACCCACAGTGATGCTTAGGGAAAGACCCTGCTCAGGACCCAGCAGGTCAGCACCCCAGAGCAGACTGATAGGTCCGTGGGACCCATGTTAGAGCAGAAAATTTGGGCTCAGCACATTTTACTGTTAGTAGAGAGCCAGGAAACGTTTTCTGGGTTGGGGATTTTGTGGGATTTTTTAATTTTTTTAGTAGGTTTTGTTTAACCTCTGTGCAGTTTGTATGAATGAATTGCTATACATTTATAAGGAGCCAGGGTCTGGAGGGTTGCTATCACTTTGTCCAGCCCAAATACCTTCCTGGGCAACTCCTACCATTTGTTTGCAGTTGCCT3

Luciferase assays were performed as described previously [3]. In short, HeLa cells were seeded in 24-well plates in light (L) SILAC medium (1×10^5^cells/well) the day before transfection which guaranteed a confluence of 90% the next day. The Fkbp8 or Prkar2a luciferase reporters were transfected into HeLa cells together with the respective miR-34 members and the pGL3 control vector (Promega) using Lipofectamine 2000 (Invitrogen) according to the manufacturer’s instructions. For the pGL3- Vcl reporter we used pRL-TK as a control plasmid (Promega). For transfection 180 ng of the reporter, 20 ng of control plasmid and 100 nM siRNA (final concentration) diluted in serum-free DMEM were used. All transfections were done in triplicates and each measurement was done three times. miR-16 was used as control miRNA that did not affect the synthesis of the examined genes as determined by MS (data from Selbach et al., 2008). The day after transfection the medium was changed and 48h after transfection cells were prepared and measured using the Luciferase Reporter assay system (Promega) according to manufacturer’s instructions. Fluorescence was measured on a MicroLumat Plus LB 96V luminometer (Berthold Technologies) and processed using MikroWin 2000 (Mikrotek Laborsysteme GmbH). Renilla luciferase activity of the reporter constructs was normalized using the activity of the firefly luciferase of the pGL3 control plasmid (Promega) (or vice versa for the Vcl reporter). Evaluation of the measurement error was done by calculating the relative error of the three biological replicates of the respective reporter along with its control and adding it up according to the law of error propagation. The relative error was used as base for computing absolute errors of the normalized expression values. To assess the pSILAC error, the standard deviation of two replicates of the miR-34a transfections (miR-34a1 and miR-34a2.1) was used. Errors are displayed as +/– two standard deviations.

### Data analysis

All data analysis was done using perl or R scripts, including spearman correlation coefficients (pairs), correlation plots, cumulative distributions (ecdf) and hypergeometric tests (dhyper). Seeds to miR-34 and * strand seeds were annotated using an in-house perl script based on 3′ UTR sequences in downloaded from the UCSC Genome Browser (http://genome.ucsc.edu, gene list update from February 2009). Input for “Sylarray” analysis (http://www.ebi.ac.uk/enright-srv/sylarray/)[42] were Refseq NM identifiers of one transfection experiment sorted from down- to up-regulated together with a background of all human gene 3′UTR sequences downloaded from the UCSC Genome Browser (http://genome.ucsc.edu, gene list update from February 2009). The options “use all available words” and “use non-redundant sequences” were selected. For gene ontology analysis and clustering we produced lists of Refseq NM Ids according to the different conditions tested as input for the online David Gene Ontology tool (http://david.abcc.ncifcrf.gov/). Refseq NM Identifiers of all proteins identified in our experiments were used as background for enrichment calculation. Output KEGG and GO biological process (GO_BP) terms were downloaded and only terms with at least p < 0.05 and 3 gene counts in one of the input datasets were used for comparison. Log2 fold changes for miR-34a expression in SW480 were extracted from Kaller et al., 2011 and mapped to our data via IPI identifiers using R. Mature miR-34 and *strand seeds were mapped. Proteins sorted according to the requirement given in the respective columns are marked with “True” or “False” if they comply with the requirement. We did not filter for * strand seeds for this analysis as this would reduce the number of shared proteins considerably.

The significance of the differences in Spearman rank correlation coefficients was computed using the Fisher r-to-z transformation with an online tool (http://vassarstats.net/rdiff.html). We treated Spearman coefficients as though they were Pearson coefficients since this procedure is more robust with respect to Type I error than either ignoring the non-normality and computing Pearson coefficients or converting the Spearman coefficients to Pearson equivalents prior to transformation [43].

## Results

### Experimental setup

**Figure 1:**
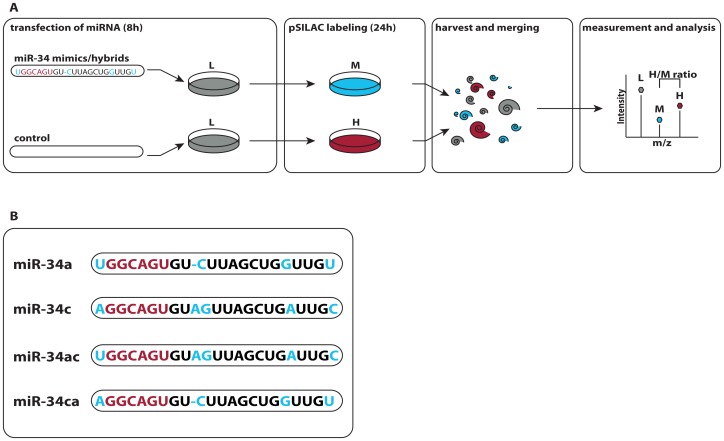
Experimental setup. **A,** Each member of the miR-34 family is transfected individually into HeLa cells in light SILAC medium. In parallel, a mock transfected control sample is prepared for each member. After 8h of transfection the samples are transferred to different SILAC medium, heavy (“H”) for the control and medium-heavy (“M”) for the miRNA transfected cells. After 24h of pulse labeling corresponding sample are combined and processed for mass spectrometry. The resulting peaks for one peptide are shown as an example. Peptides produced before pulse labeling appear as light peaks and can be disregarded. Differences in protein synthesis between control and miRNA-transfected samples can be read from the H/M ratio of the respective peptides. **B**, Nucleotide sequences of the miR-34 family members miR-34a and miR-34c. To investigate the importance of 5′ versus 3′ends two miRNA chimeras were constructed swapping head (nt 1-9) and tail of miR-34a and miR-34c respectively. Differences in the nucleotide sequence are marked in blue. The seed is labeled red.

Transfection of HeLa cells was performed using double-stranded RNAs mimicking miR-34a and miR-34c in a pulsed SILAC (Stable Isotope Labeling of Amino Acids in Cell Culture) approach as described before [3], [34], [44]. To enable measurement of changes due to miR-34 over-expression, it was ensured that none of the miR-34 members is detectably expressed in HeLa cells [45]. Double-stranded RNAs were designed as mature miR-34 mimics, 22–23nt in length and with the 3′strand designed as perfect complement to the mature 5′strand. A mock transfection control was prepared in parallel for each miR-34 transfected sample. Cells were cultivated on light SILAC medium and transfected with the miRNA via Dharmafect1. 8h after the transfection, we transferred the miRNA transfected cells to medium-heavy (“M”) and control cells onto heavy (“H”) SILAC medium, incubated for another 24h hours and subsequently harvested them. Differentially treated cells were combined with the mock control and analyzed by high resolution LC-MS/MS **(** FIG 1A **).** A transfection efficiency of over 90% in HeLa cells was determined using fluorophore-conjugated dsRNA prior to the experiment (FIG S1).

To control for biological and technical variability we performed biological replicates of the miR-34a and miR-34c transfection experiments on a different day in a completely independent manner. In addition, one miR-34a transfection experiment was performed twice on the same day to have a comparison of biological replicates from both the same and different days. We also designed two chimeras of miR-34a and miR-34c comprising the first nine nucleotides of the 5′end of either miR-34a or miR-34c paired to the remaining 3′end nucleotides of the respective other miRNA. The two chimeras miR-34ac and miR-34ca (first letter indicates parent 5′, second parent 3′ end) were processed the same way as the miR-34 members. Sequences of all miR-34 family members and chimeras can be seen FIG 1B.

### miR-34a and miR-34c induced changes in protein synthesis

**Figure 2:**
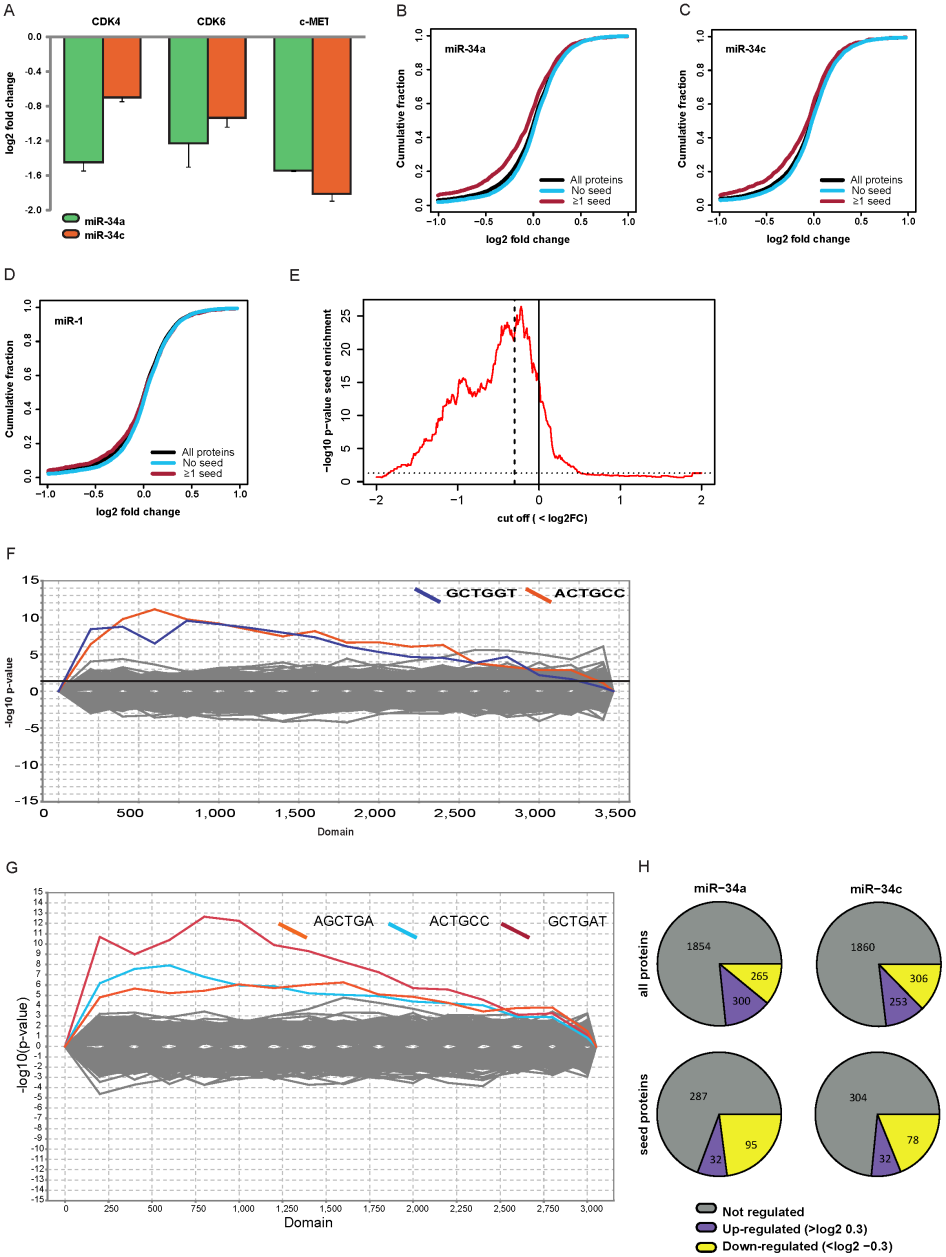
MiR-34a and miR-34c repress synthesis of many proteins. (**A**) Known targets of the miR-34 family are down-regulated in our dataset (error bars indicate standard deviations from two or three experiments). (**B**) Cumulative distribution plots show that synthesis of proteins with miR-34 seed matches in their mRNA 3′UTRs is repressed by transfecting miR-34a (n = 4612). (**C**) The same holds true for the miR-34c transfection (n = 4094). (**D**) When selecting for the seed of miR-1 this correlation between seed and down-regulation is not visible (n = 4612). (**E**) Enrichment of seed matches in down-regulated proteins is significant even at mild log2FC cut-offs (hypergeometric test, dashed line: log2FC cut-off -0.3, dotted line: significance threshold p = 0.05, n = 4612). (**F**) Sylarray analysis [42] of miR-34a proteins sorted from down- (left) to up-regulated (right) renders the mature miR-34a seed (ACTGCC) as enriched nucleotide motif; however, also the *seed of our siRNA duplex (GCTGGT) is enriched in down-regulated proteins**.** (**G**) Similar observations are made for miR-34c. (**H**) Overview of the numbers of quantified as well as regulated proteins in miR-34a and miR-34c.

Mass spectrometry lead to the identification of overall 6,241 and quantification of 5,435 proteins in all experiments. We required at least two quantified peptide evidences in each experiment, resulting in about 2,400 to 4,800 quantified proteins in each individual transfection experiment at a false discovery rate of 1%. The complete set of quantified proteins is given in Table S1. Several targets of the miR-34s described in literature were down-regulated in our data as well (FIG 2A). Note, that we removed all proteins that we were not able to map to a specific mRNA from all further analysis.

Since the seed match within 3′UTRs is important for miRNA target selection, direct miR-34 targets should be enriched for 3′ UTR seeds. We therefore investigated if proteins with a seed match in their corresponding mRNAs are down-regulated at the protein level. This can be visualized using cumulative distribution plots of the miR-34 transfections. Here, the distribution of log2 fold changes for proteins with or without a 3′UTR seed match are plotted. “Seed proteins” clearly showed reduced protein synthesis compared to non-seed proteins (FIG 2 B,C). This effect is not observed with a seed of a different miRNA such as miR-1 (FIG. 2D). We conclude that many proteins in our pSILAC data are directly repressed by miR-34.

Next, we sought to determine a cut-off value to define targets of miR-34. To this end, we calculated how significant the enrichment of proteins with 3′ UTR seed matches is at different cut-offs using the hypergeometric test (FIG 2E). Proteins down-regulated by miR-34a were highly significantly enriched in miR34 seeds even at mild cut-offs (for example, p = 6×10^−16^ for proteins with log2FC < 0). To minimize false positives we used a more stringent cut-off of –0.3 (p = 3.8×10^−23^, dashed line). To obtain an estimate of the actual number of direct targets identified at this cut-off we asked how many of the down-regulated proteins can be explained by the seed. 655 and 687 proteins had a log2FC smaller than –0.3 in the miR-34a and miR-34c experiments, respectively. Of these down-regulated proteins, 275 (42%) and 257 (37%) had a 3′ UTR seed match for miR-34a and c. The background seed frequency of non-regulated proteins (absolute log2FC<0.1) was 23% in both cases. Therefore, about 19% (miR-34a) and 14% (miR-34c) of down-regulated proteins with a seed match are expected to be direct targets. This amounts to 52 targets for miR-34a and 36 for miR-34c. It should be noted that these estimates only include targets with 3′ UTR seed matches. Seed matches in the coding sequence or targets without seed matches are not included. Thus, the true number of direct targets is probably higher.

A nucleotide motif enrichment analysis employing the online tool “Sylarray” [42] revealed that not only the signal for the mature miRNA but also the *strand seed of the respective miR-34 member was detectable (FIG 2F,G). Recent studies suggest that the incorporation of the *strand seed might be a common trait for miRNAs and physiologically important [46], [47], [48]. However, since the transfected RNAs were designed as perfect duplexes, the sequence of the *strand we used in our experiments differs from the endogenous version, most notably in the *seed region. To minimize the impact of the artificial *seed in our data we excluded all proteins with any of the *seed sequences in their 3′UTRs. This reduces the number of quantified proteins to 2419 in the miR-34a and miR-34c transfection experiments (1204 proteins in all replicates). FIG. 2H gives an overview of the regulation of proteins by miR-34a and miR-34c. Table S1 shows all quantified proteins and mRNA abundance for the miR-34 transfections for genes not containing a *strand seed site in their 3′UTR. Further data analysis was done using the two miR-34 experiments and the 2419 proteins quantified unless stated otherwise.

### Correlation and differences in protein regulation by miR-34a and miR-34c

**Figure 3:**
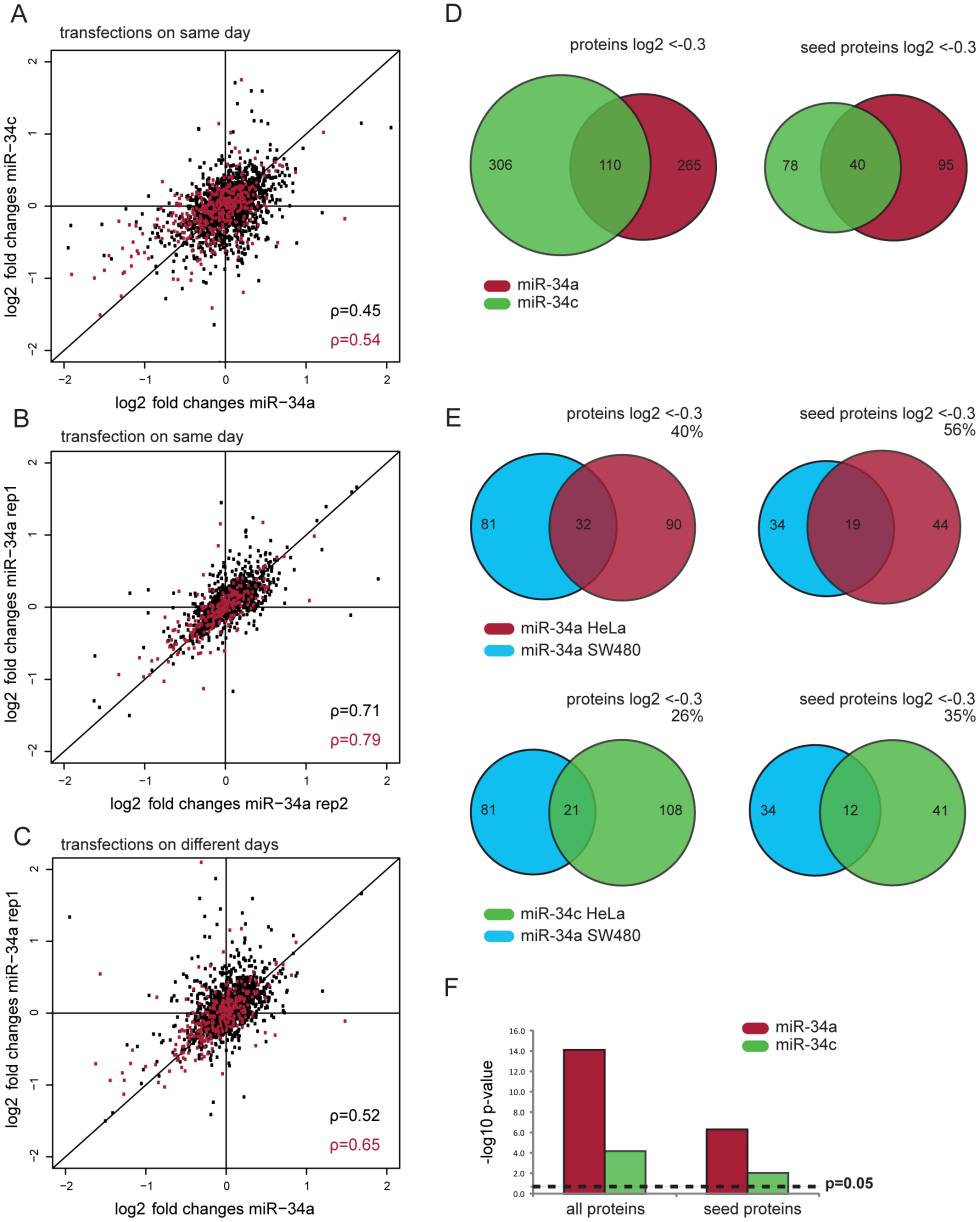
Proteomic comparison of miR-34a and miR-34c targets. (**A**) The correlation of log2 fold changes between miR-34a and miR-34c in the same transfection experiment (n = 2419) show a lower Spearman correlation than the two replicates of miR-34a (n = 1404) (**B**). This holds also true when comparing miR-34a experiments from different days (n = 1777) (**C**). Spearman coefficients for all proteins are marked in black, while seed containing proteins are indicated in red. (**D**) The overlap of common targets between miR-34a and miR-34c is rather low. (**E**) The overlap of miR-34a targets (–0.3 log2 FC) from SW480 cells [29] is bigger with miR-34a than with miR-34c targets in our HeLa dataset. Venn diagrams show the overlap of the 81 down-regulated proteins quantified in both the Sw480 and our HeLa dataset. Numbers in Venn diagrams depict total number of proteins down-regulated by log2 < –0.3 for one miR-34 or shared by two miR-34 members. The percentage of down regulated SW480 proteins that are also down regulated in HeLa cells is given above the diagrams. (**F**) The overlap with miR-34a targets in SW480 cells is more significant for miR-34a than for miR-34c in HeLa cells (hypergeometric test).

Next, we compared pSILAC data for miR-34a and miR-34c. Log2 fold changes for both miRNAs were clearly correlated (FIG 3A, rho  =  0.45). However, the scatter is higher than in typical biological replicates with the same miRNA, suggesting that targets of both family members are overlapping but not identical. To assess the experimental variability in our data we performed two parallel miR-34a experiments. Indeed, these experiments showed considerably higher correlation (FIG 3B, rho  =  0.71). Of note, even two miR-34a experiments performed on different days correlated better with each other than the miR-34a and miR-34c data derived from parallel experiments on the same day (FIG 3C). Next, we computed whether the observed differences in Spearman correlation coefficients are statistically significant using the Fisher r-to-z transformation [43]. We found that the correlation between miR-34a and miR-34c is significantly lower than the correlation between two miR-34a replicates performed on the same day (p < 0.0001). Even the correlation of miR-34a replicates performed on different days is significantly better than the correlation between miR-34a and miR-34c (p ≤ 0.0017). This analysis strongly suggests that the impact of both family members on protein synthesis is not identical. Interestingly, miR-34a and miR-34c display the biggest differences in down-regulated proteins, a hint that they might mainly differ in their putative direct targets. In fact, less than half of the seed proteins which are down-regulated by miR-34a are also down-regulated by miR-34c (log2FC < –0.3, FIG 3D). This indicates that despite the similarities between miR-34a and miR-34c on protein regulation, each family member down-regulates a distinct set of putative targets. To minimize the impact of biological variability we focused our subsequent analysis on the miR-34a and miR-34c experiments performed on the same day.

On one hand, transfecting cells with dsRNAs mimicking mature miRNAs is advantageous since it avoids possible differences in miRNA processing between family members. On the other, our artificial over-expression system might also induce unspecific effects. We therefore compared our data with results obtained by expressing the full precursor of miR-34a in SW480 cells [29]. In this study, an episomal pRTS-miR-34a plasmid was induced for 16h and pSILAC labeled for 24h. Of the 1,206 quantified proteins in this study 946 could be mapped to our HeLa dataset (of which 212 have a seed match). 81 of the shared proteins were down-regulated in SW480 cells (log2 fold change < –0.3). Among these, 32 and 21 were down-regulated by miR-34a and miR-34c in our HeLa data, respectively (FIG 3E). Hence, miR-34a in SW480 cells shares more potential targets with miR-34a than with miR-34c in HeLa cells. This observation also holds true when only proteins with a seed match in their 3′UTR are considered. The overlap for miR-34a in both datasets is highly significant with p-values of 8.4*10^−15^ and 5.3*10^−07^ for non-seed and seed proteins, respectively (hypergeometric test, FIG. 3F). For miR-34c the overlap is less significant (p = 7.4*10^−05^ and p = 0.009). As a control, we also compared the overlap of our miR-34 data with pSILAC data obtained for a different miRNA (miR-1, dataset taken from Selbach et al., 2008). In this biologically unrelated control the p-values exceed the significance threshold (p = 0.15, data not shown). The fact that the overlap for miR-34a is considerably higher than for miR-34c indicates that the observed differences between miR-34a and miR-34c are not an artifact of our experimental approach. Instead, the observation that our data can be replicated in a different cell type by a different group, using a different miRNA expression system and different pulse labeling times strongly suggests that results are robust and meaningful beyond our specific experimental conditions. In addition, it is in accordance with our observation that miR-34a and miR-34c down-regulate different sets of targets.

### Pathway analysis of miR-34a and miR-34c affected proteins

**Figure 4:**
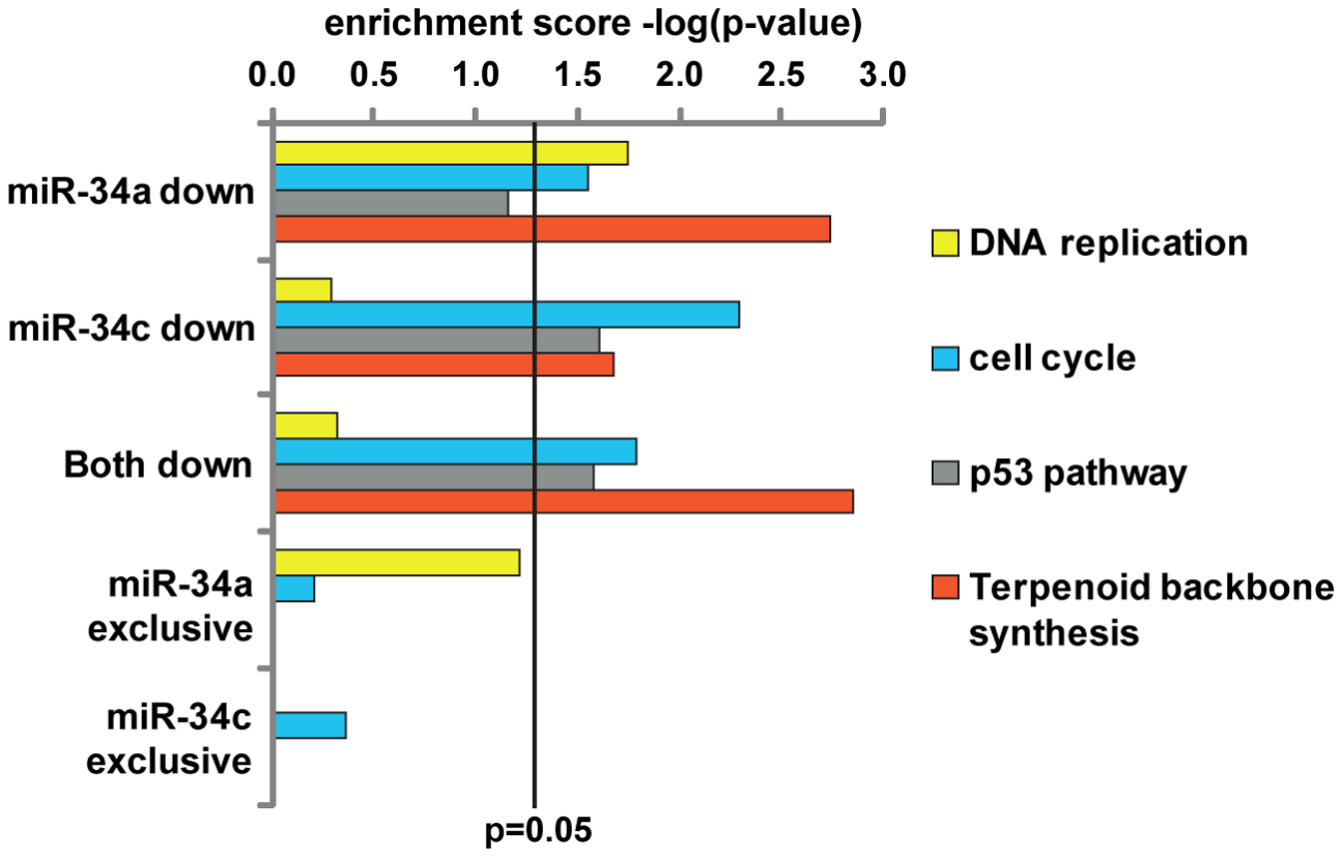
Functional enrichment analysis of miR-34a and miR-34c. KEGG pathway enrichment for subsets of miR-34a and miR-34c targets (for all proteins down-regulated log2 < -0.3 by one member, shared by both and exclusive targets of one miR-34 member). Enrichment is depicted as the -log of the respective p-value of the enriched term.

Having shown that miR-34a and miR-34c have overlapping but not identical targets, we next asked if these differences might reflect different biological functions. We therefore employed the online DAVID gene ontology tool to look for enriched KEGG pathways. Both miRNAs affected the pathways “cell cycle”, “p53 pathway” and “terpenoid backbone synthesis” (“both down”, FIG. 4). Hence, pathway analysis indicates functional redundancies of both miRNAs. One pathway mostly enriched in miR-34a is “DNA replication” which includes the Mcm proteins (Mcm3/5/6/7) and Pold1 (DNA polymerase delta catalytic subunit 1). The miR-34a specific enrichment is also visible when only exclusive targets of the two miRNAs are considered (miR-34a and miR-34c exclusive, Fig. 4). Some of the involved proteins are also mildly regulated by miR-34c which actually has been reported to regulate “DNA replication” as well [33]. However, in our study miR-34a outnumbers miR-34c concerning targets in this KEGG pathway. In addition, important genes necessary for nucleotide synthesis and thus DNA replication such as Impdh1 are specifically repressed by miR-34a. In summary, comparison at the level of pathways suggests overlapping functions with a seemingly stronger impact of miR-34a on “DNA replication”. More details about pathway enrichment and names of corresponding proteins are available in Table S2.

### miR-34a and miR-34c chimeras exhibit specific 5' or 3′end co-regulation of exclusive targets-

**Figure 5:**
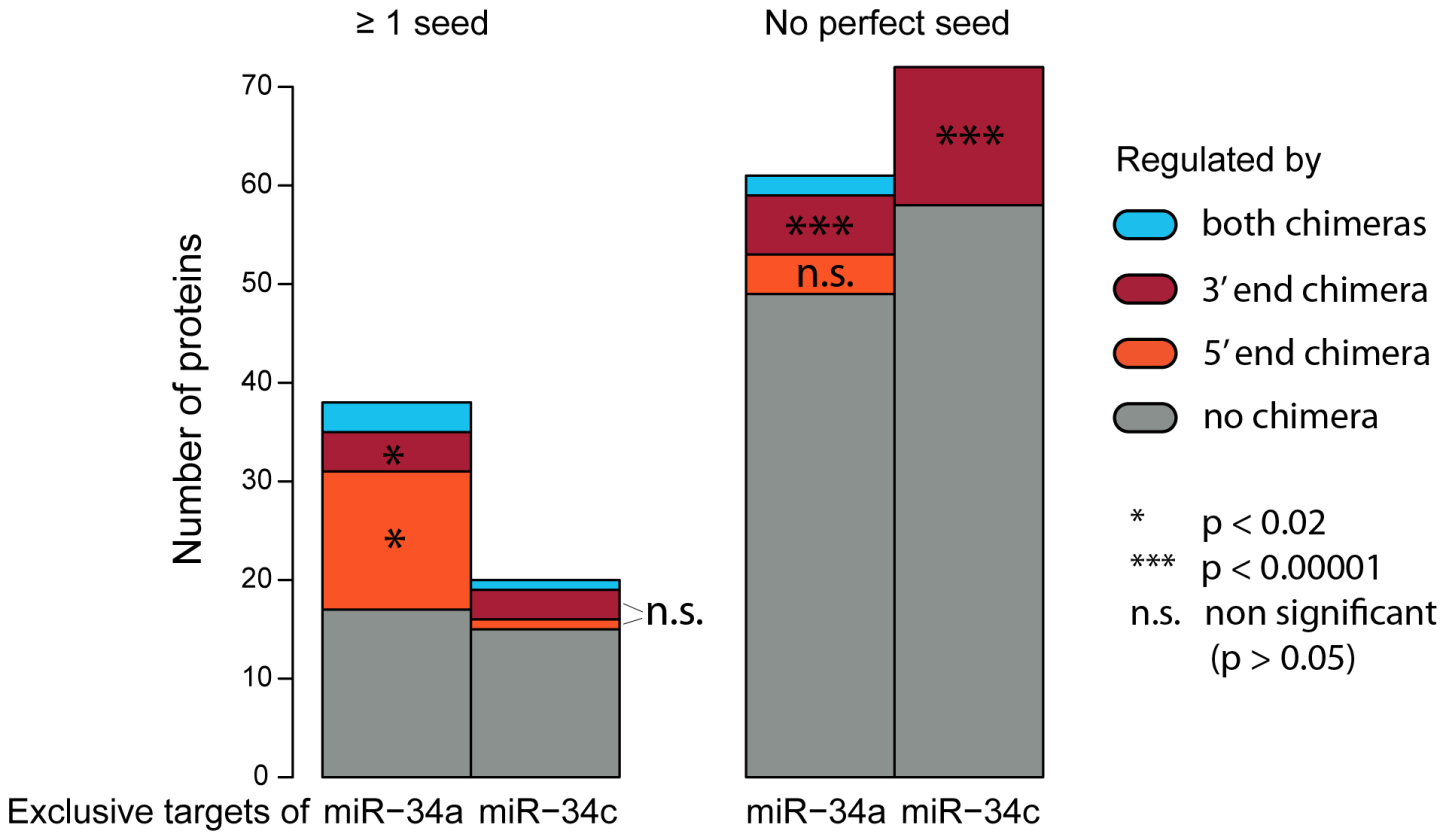
Distribution of chimera regulation within exclusive targets for either miR-34a or miR-34c. Shown are proteins exclusively regulated by miR-34a or miR-34c with a log2 fold change cutoff of ≤ –0.3. Colors indicate whether these exclusives targets are also regulated by any or both miR-34 chimeras mir-34ac and miR-34ca. The significance of the overlap between exclusive targets of the miRNAs and their chimeras was calculated using a hypergeometric test.

To assess sequence specificity in target selection we compared which exclusive targets of miR-34a or miR-34c were down-regulated in their respective 5′ or 3′end chimera (FIG. 5). The exclusive targets of both miR-34 members are only partly co-regulated by the chimera miRNAs, especially for targets without a seed site. This is expected since genes lacking 3′ UTR seeds are more likely indirect targets. Interestingly, miR-34a and miR-34c show different chimera preferences. For seed containing targets, the 5'end chimera of miR-34a (mir-34ac) shows stronger and statistically significant co-regulation of targets. miR-34c, however, does not exhibit this effect. In addition, miR-34c has a much smaller number of exclusive targets containing a seed site than miR-34a. Taken together, this indicates that some of the specific target selection of miR-34a might be due to its 5'end with the first nucleotide being an uracil as opposed to an adenine for miR-34c. For targets without a seed site however, a clear bias towards a co-regulation by the 3'end chimera of miR-34c is visible. Thus for miR-34c, this implies that base pairing with the 3′end of the miRNA governs target recognition in the absence of a seed as has been suggested for individual examples before [14]. Importantly, for targets without a perfect seed site the overlap with the 3′ end chimera-specific targets is highly significant for both miR-34a and miR-34c. Hence, the data from our chimera experiments is consistent with the idea that specificity is mainly determined by the 3′ end of the miRNA in the absence of a seed. If this 3′end binding requires an imperfect seed site or is sufficient for down-regulation on its own cannot be concluded from this analysis. We analyzed our data using RNAchimera [49] to search for sequence motifs associated with the minimum hybridization energies of mRNA and miR-34 members. However, no specific sequence motif could be clearly associated with the co-regulation of 3'end or 5'end chimera regulation of exclusive targets. Nevertheless, the fact that miR-34a and miR-34c show opposite biases for chimera co-regulated targets clearly suggests that their sequence might be important for a target-based distinction between both miR-34 members.

### Verification of specific targets of miR-34a and miR-34c

**Figure 6:**
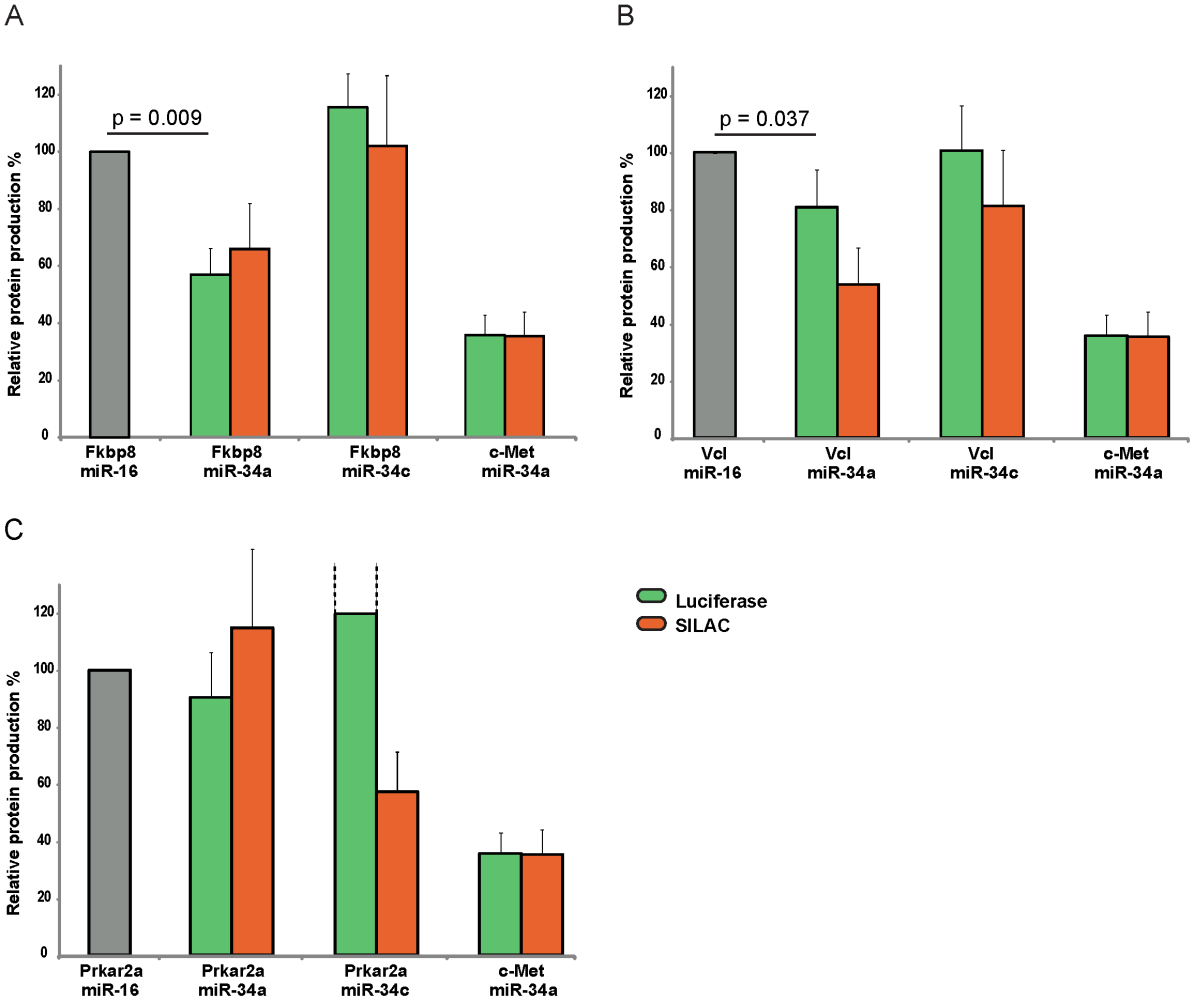
Luciferase assays of specific miR-34a and miR-34c targets. Displayed is the relative protein production after transfection of miR-34a and miR-34c together with vector constructs containing the 3′ UTR of the two seed-containing miR-34a specific targets Fkbp8 (**A**) and Vcl (**B**) or the seedless miR-34c specific target Prkara2a (**C**). The SILAC change displays the difference of log2 fold changes as observed in the proteomic data. The known miR-34 target c-Met is used as control vector and miR-16 as control siRNA that does not significantly influence the levels of either target. Relative protein production for Prkar2a transfected with miR-34c were higher than depicted, reaching a 191%, which is indicated by dashed lines. P-values were calculated by one-tailed one-sample t-test from n  =  3 biological replicates.

Collectively, our results suggest that miR-34a and miR-34c have both shared and unique targets, and that some unique targets can only be observed at the protein level. To validate our findings we selected three unique targets from our pSILAC dataset for validation by luciferase assays. To create a reporter construct we fused the full length 3′ UTR of the targets Fkbp8, Vcl and Prkar2a to the coding sequence of luciferase. We then co-transfected the constructs with miR-34a and miR-34c and quantified protein production by luciferase assays (FIG. 6). As a positive control we used the 3′UTR of c-MET, a known target of the miR34 family [50], [51].

In our data FK506-binding protein 8 (Fkbp8) is repressed at the protein level in all three miR-34a experiments but unchanged in both miR-34c experiments. Neither miR-34a nor miR-34c had a significant impact on mRNA levels of Fkbp8 (data not shown). Hence, Fkbp8 might be a miR-34a specific target regulated mainly at the protein level. Fkbp8 acts as a chaperone which stabilizes the anti-apoptotic protein Bcl-2 and thereby contributes to tumorigenesis and chemoresistance [52], [53], [54]. The 3′ UTR of the Fkbp8 mRNA has a single seed match to miR-34. We found that miR-34a but not miR-34c significantly inhibited the Fkbp8 luciferase construct (FIG. 6A). Moreover, changes quantified by luciferase assays were overall very similar to changes quantified by pSILAC. The same result can be seen for the miR-34 seed containing Vinculin (Vcl), a protein important for cell structure and adherens junctions (FIG. 6B). Both SILAC and luciferase data show a stronger regulation of Vcl by miR-34a and a lesser or no regulation by miR-34c. While both methods share the same pattern, the regulation of Vcl turns out milder in the luciferase assays for both miR-34a and miR-34c. We also assessed the regulation of one miR-34c specific target without a seed site. Prkar2a is the regulatory subunit of protein kinases involved in cAMP-dependent signaling. It activates protein kinase A which in turn phosphorylates many substrates with multiple functions in the cell, such as sugar, glycogen and lipid metabolism. Down-regulation of Prkar2a is associated with cell growth inhibition and tumor suppression [55], [56], [57]. Our SILAC data indicate that it is exclusively down-regulated by miR-34c. However, luciferase assays could not replicate the down-regulation by miR-34c but show a clear up-regulation of Prkar2a instead (FIG. 6C). This result indicates that Prkar2a is in fact not regulated by miR-34c via its 3'UTR and thus probably no direct target. These results validate pSILAC data for Fkbp8 and Vcl and demonstrate that the 3′ UTR is sufficient for the observed down-regulation by miR-34a. Collectively, this suggests that both proteins are direct targets of miR-34a but not miR-34c. The results for Prkar2a however, suggest that some observed exclusives targets without a seed site might not be directly regulated by miR-34c.

## Discussion

Few microRNA families have been investigated for their differential targeting in microarray studies before with the conclusion of their functional redundancy [18]. However, no large scale study of protein levels has been done up to date. Here, we provide insights into common and differential targeting of the miR-34 family members miR-34a and miR-34c which play a pivotal role in tumor progression in many cancer types [23], [32]. We observe that targets of both family members overlap significantly. Thus, our proteomic data is overall consistent with previous conclusions based on microarray experiments [18]. However, we also provide evidence suggesting that targets of both family members are not entirely redundant: First, we observe that the difference between pSILAC log2 fold changes induced by miR34a and miR34c is higher than expected compared to replicate experiments with the same miRNA. Second, we find that miR-34a targets identified in a different cell line using a different expression system show a higher overlap with miR-34a than with miR-34c targets we identified. Third, pathway analysis indicates differences in the cellular function of both family members. Finally, luciferase assays for Fkbp8 and Vcl support that these genes are exclusive targets of miR-34a.

It should be stressed that not all differences in the pSILAC data result from direct effects of the transfected miRNAs. For example, direct targets may in turn have secondary effects on protein production. We expect that some of the observed differences are caused by such indirect effects, even though we tried to minimize them by performing pSILAC measurements shortly after transfection. Moreover, our data is affected by technical and biological variability. Thus, not all of the proteins that seem to be differentially affected by both family members will be truly differential targets. Table S1 provides pSILAC data for individual experiments and can therefore be used as an additional reliability filter. It is also possible that the efficiency of incorporation into the RNA-induced silencing complex (RISC) differs between family members. However, this is unlikely in our experimental set-up since we used over-expression and observed overall similar repression of the known targets Met, Cdk4 and Cdk6 at both the protein and mRNA level.

Another concern is that over-expression of miRNAs could lead to unspecific selection of targets that would not be repressed by physiological concentrations of the miRNA [58], [59]. However, it has been reported that over-expression and knock-down of an individual miRNA induces anti-correlated changes in protein synthesis[3]. Thus, targets down-regulated by miRNA over-expression are relieved of repression and up-regulated to a similar extent in miRNA knock-down. If this observation also holds for miR-34 is however not clear. Non-physiological targeting is a risk inherent in the model of miRNA mis-expression. For example, expression of miRNAs in cell lines and tissues outside their physiological context might identify targets that are physiologically irrelevant. This may also be important for miR-34 since family members are expressed in a tissue specific manner [23]. While miR-34a is ubiquitously expressed, miR-34b and miR-34c are of very low abundance except for lung tissue where they outnumber miR-34a.

Luciferase assays are one option to further validate differential targeting and to test if repression depends on signals in 3′ UTRs. Data from luciferase assays for the seed match containing 3′ UTRs of Fkbp8 and Vcl was consistent with pSILAC results, indicating that at least some of the observed differences are indeed due to the different family members. On the other hand, the 3′ UTR of Prkar2a lacks a seed match and is not repressed by miR-34c in the luciferase assay, even though pSILAC suggests Prkar2a as a miR-34c specific target. Whether this discrepancy is due to 3 ′UTR independent direct repression, indirect repression via other miR-34c targets or measurement noise cannot be assessed at the moment.

Base pairing between the miRNA seed and complementary sequences in 3′UTRs is generally considered to be an important factor in target recognition, although examples of targets without a perfect seed match also exist. We observed that down-regulated proteins are clearly enriched for 3′UTR seed matches, confirming the importance of the seed. Interestingly, proteins with seed matches showed a higher correlation between both miR-34a and miR-34c than proteins without a perfect seed match. This might suggest that differential targeting is more prominent for targets without a perfect seed match. Alternatively, it is also possible that differences in proteins lacking a seed match are due to indirect effects. In either case, our data for Fkbp8 and Vcl implies that also proteins with seed matches can be differentially targeted. This is in line with the observation that c-Myc is preferentially targeted by miR-34c [33]. Another important finding was the observation that not only the seed of the mature miRNA but also the *strand seed of miR-34a and miR-34c affected protein abundance in our data. We therefore excluded proteins containing a *strand seed site in our analysis. The *strand of endogenously expressed miRNAs can be physiologically relevant [48], [60], [61]. Analysis of *strand activity is often omitted in studies and retrospective data analysis of a retroviral expression study of miR-34 in HCT116 cells [62] provided evidence that the *seed was visible in the data [48]. However, in cases were miRNA mimics are designed as perfect siRNA duplexes the observed *seed does not necessarily match the endogenous seed. This is an important consideration for data interpretation: The impact of the * stand limits our ability to unambiguously identify miR-34a or miR-34c specific targets. While we excluded all genes with perfect matches to the * strand seed from our analysis, we cannot exclude indirect and/or seedless targets of the artificial * strand. Thus, some of the observed differences between miR-34a and miR-34c might in fact be mediated by the * strand. Having said this, the highly significant overlap with results obtained by expressing the miR-34a precursor strongly suggests that our data is meaningful beyond our specific experimental conditions. Further experiments to directly compare the endogenous precursors of miR-34a and miR-34c will help clarifying this point.

To analyze the influence of 5′ and 3′end differences on target selection, we also transfected chimera miR-34s comprising a mixture of the 5′end of one with the 3′end of the respective other miR-34 member. Seed containing exclusive targets of miR-34c displayed an equal distribution of co-regulation with its 3′end or 5′end chimera. The higher co-regulation of exclusive miR-34a targets by its 5'end chimera, however, suggests that the influence of the first miRNA nucleotide might be important for the target selection of miR-34a. Exclusive targets of both miR-34a and miR-34c on the other hand showed a strong co-regulation with its respective 3'end chimera, suggesting that 3′end binding might mediate this repression. This is consistent with earlier studies on target selection of miRNA families which suggested 3′end supplementary pairing as the reason for member specific targeting in case of an imperfect seed site [14], [63]. Thus the influence of 3′end complementing imperfect or absent seed sites should not be underestimated in miRNA targeting.

**Figure 7:**
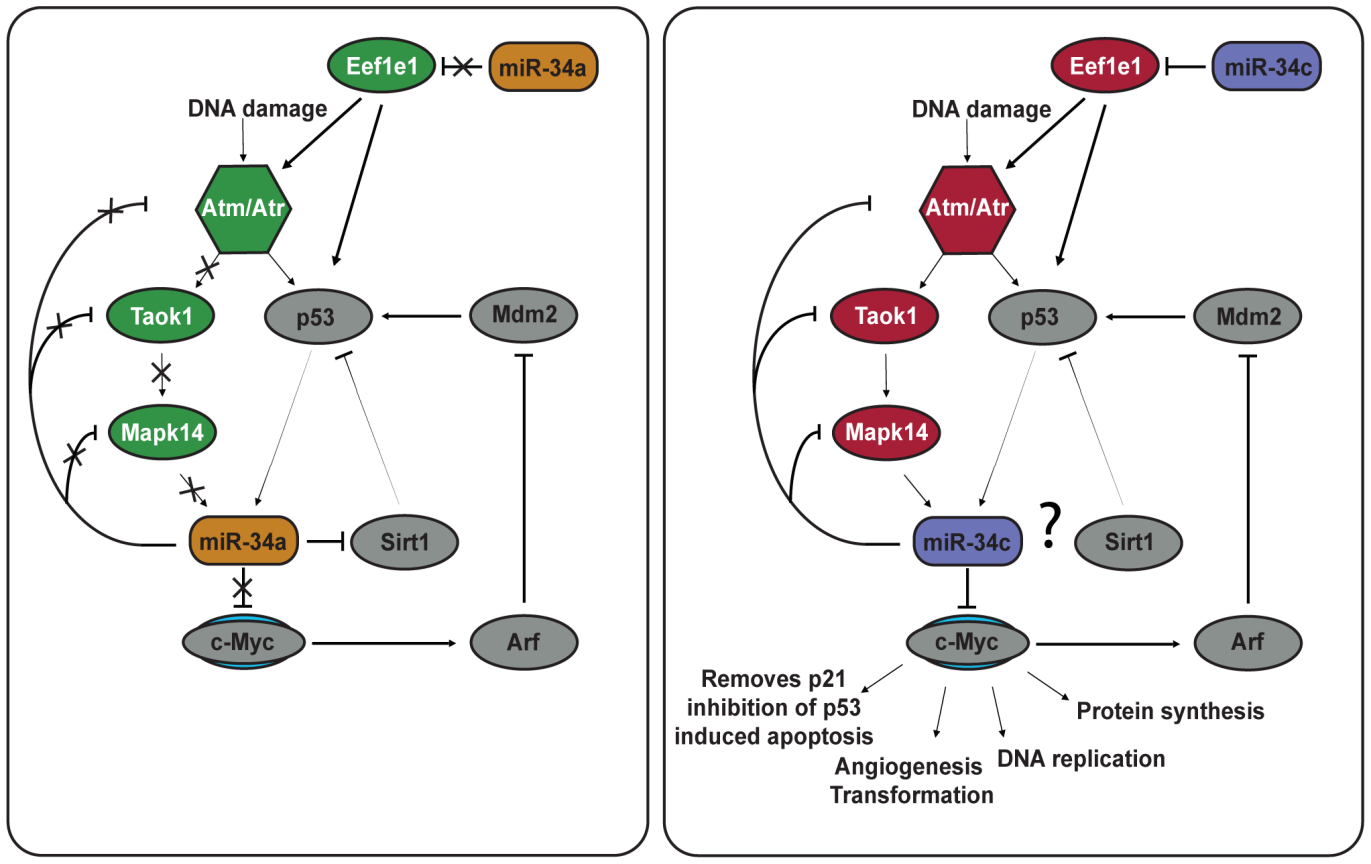
Hypothetical model of the differential effect of miR-34a and miR-34c on the p53 feedback network integrating both known protein interactions with miR-34a and miR-34c and our observations. Grey color indicates proteins not quantified in our data but with known miR-34 interactions. Color indicates down-regulation (red) and up-regulation (green) of proteins quantified in our data. miR-34a has a positive feedback loop with p53 by blocking its inhibitor Sirt1. The effect of miR-34c on Sirt1 is not known. While miR-34a induction is heavily dependent on p53 levels, miR-34c expression can also be induced via alternative pathways (of which Mapk14 is depicted here). c-Myc is no target of miR-34a under normal expression conditions but is strongly repressed by miR-34c. This leads to inhibition of cell proliferation, DNA replication and induction of S-phase arrest. c-Myc also hinders apoptosis induction under p53 activation settings.

Our data provides a resource for the scientific community that might be useful to unravel the functions of the miR-34 family. Besides cell cycle arrest and DNA damage repair, miR-34 induction via p53 can also lead to senescence and apoptosis [28]. We observed that miR-34a down-regulates a number of anti-apoptotic targets such as Gclm, Hspa1a and most importantly Fkbp8. The latter directly regulates levels of Bcl-2 by acting as a chaperone, and down-regulation of Fkbp8 leads to apoptosis [53], [54]. Fkbp8 has further functions in regulation of cell cycle progression and cancer by triggering the degradation of Prl-3 via the 26S proteasome [64]. miR-34c on the other hand, targets several pro-apoptotic genes such as Pkn2, Eef1e1 and Taok1. It is tempting to speculate that miR-34a is overall more pro-apoptosis than miR-34c (see Fig. 7 for a hypothetical model). While further experiments are clearly needed to address this point, it is in fact consistent with previous reports: Apoptosis seems to depend on a miR-34a mediated positive feedback loop that amplifies p53 activation [62], [65]. miR-34a amplifies p53 levels by targeting Sirt-1 [66]. In addition, only miR-34c down-regulates c-Myc under normal expression conditions [33]. While elevated levels of c-Myc lead to p53 amplification and apoptosis, down-regulation inhibits apoptosis and DNA replication followed by S-phase arrest [33]. We neither detected Sirt-1 nor c-Myc in our proteomic data. However, our observation that the important p53 effectors Eef1e1, Atm, Taok1 and Mapk14 are exclusively down-regulated by miR-34c complements previous findings: Eef1e1 is the key up-stream activator of Atm/Atr and the repression of both leads to lower p53 levels [67]. Similarly, the miR-34c levels are reduced by down-regulation of Taok1 which phosphorylates Mapk14, a kinase that directly regulates miR-34c levels [33], [68]. It is tempting to speculate that a main difference of the two family members is that miR-34c dampens the initial DNA damage signal while miR-34a amplifies it. Further functional studies are required to test this hypothesis. Finally, the miR-34 family has recently been reported to be also involved in neuronal and cardiovascular diseases [69], [70]. While discussing these aspects is beyond the scope of this study, it will be interesting to see if our data also suggests functions outside the cancer context.

## Supporting Information

Figure S1 **Efficiency of siRNA transfection in HeLa cells.** Fluorophore-conjugated dsRNA (“BLOCK-IT”) were transfected into HeLa cells (a) and show a clear signalfor over 90% of cells, while (b) non-transfected cells do not display fluorescence. (For details see Material and Methods).
(TIF)

Table S1 **Complete set of identified proteins.**
(XLSX)

Table S2 **Pathway enrichment analysis.**
(XLSX)
