# Supplementary figures and images for: Quantitative Proteomic Analysis of Gene Regulation by miR-34a and miR-34c
Source: PLoS One. 2014 Mar 17;9(3):e92166. doi: 10.1371/journal.pone.0092166 (PMC3956911; doi:10.1371/journal.pone.0092166)

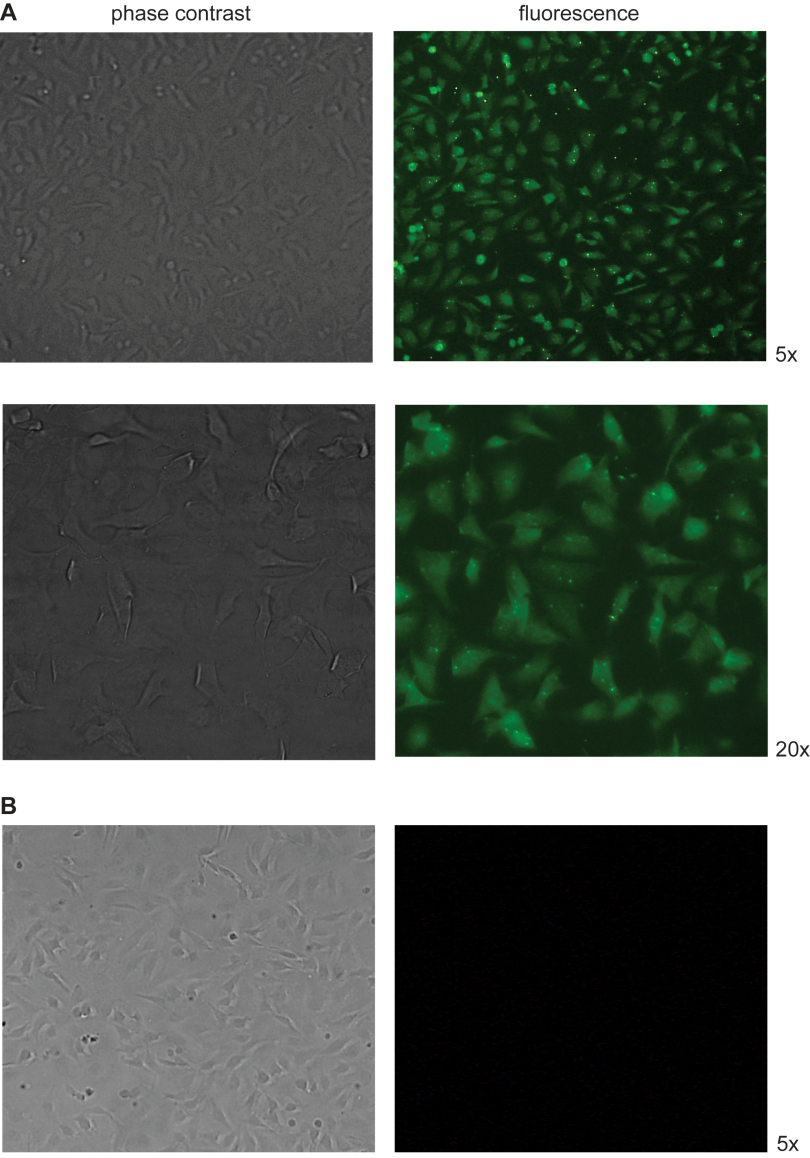

Supplement: Figure S1 — Efficiency of siRNA transfection in HeLa cells. Fluorophore-conjugated dsRNA (“BLOCK-IT”) were transfected into HeLa cells (a) and show a clear signalfor over 90% of cells, while (b) non-transfected cells do not display fluorescence. (For details see Material and Methods). (TIF) [file pone.0092166.s001.tif]
